# Recovery time and its predictors of severe acute malnutrition among under five children admitted at the therapeutic feeding center of Hiwot Fana comprehensive specialized hospital, eastern Ethiopia, 2024: a semi-parametric model

**DOI:** 10.3389/fnut.2024.1450496

**Published:** 2024-11-05

**Authors:** Fentahun Meseret, Mulualem Keneni, Ayichew Alemu, Diribsa Tizazu, Tesfaye Asfaw Alemayehu, Yalew Mossie, Tilahun Teshager, Fenta Wondimneh

**Affiliations:** ^1^Department of Pediatrics and Child Health Nursing, College of Health and Medical Science, School of Nursing, Haramaya University, Harar, Ethiopia; ^2^Department of Adult Health Nursing, College of Health and Medical Science, School of Nursing, Haramaya University, Harar, Ethiopia; ^3^Department of Emergency and Critical Care Nursing, College of Health and Medical Science, School of Nursing, Haramaya University, Harar, Ethiopia

**Keywords:** sever acute malnutrition, recovery, time, under five children, Ethiopia

## Abstract

**Background:**

Early recovery is a performance indicator of quality care for children under five admitted due to severe acute malnutrition (SAM) at therapeutic feeding centers. Despite the available interventions to tackle such nutritional problems, there is limited information on the time to recovery and its predictors among children with severe acute malnutrition in Ethiopia, more particularly in the study setting.

**Objective:**

The study aimed to assess the time to recovery from severe acute malnutrition and its predictors among children aged 6–59 months admitted to the therapeutic feeding center (TFC) of Hiwot Fana Comprehensive Specialized Hospital (HFCSH), eastern Ethiopia, from 1st September 2019 to 1st March 012024.

**Methods:**

A retrospective cohort study was conducted at the therapeutic feeding center of HFCSH among a randomly selected sample of 349 patients with severe acute malnutrition who were undergoing therapeutic feeding. Data were collected using a data abstraction tool and then stored in Epi-data version 4.6 and STATA version 17.0 statistical software. Descriptive statistics, Kaplan–Meier (KM) plots, median survival times, the log-rank test, and the Cox proportional hazards regression model were used to report the findings of this study. After performing the Cox proportional hazards regression, the model goodness of fit and assumptions were checked. Finally, the association between independent variables and the time to recovery in days was assessed using the multivariable Cox proportional hazards model, and the variables with a *p*-value <0.05 were considered statistically significant.

**Results:**

The median survival time to recovery among the patients with severe acute malnutrition was 17 days [95% confidence interval (CI): 16–18]. The incidence density recovery rate was 5.7 (95%CI, 4.9–6.6) per 100 person-days of observation. Factors that affected the time to recovery included residing in rural areas [adjusted hazard ratio (AHR) = 2.072; 95%CI = 1.336–3.215], being vaccinated according to age (AHR = 1.848; 95%CI = 1.162–2.939), and lack of analgesic administration (AHR = 0.685; 95%CI = 0.472–0.995).

**Conclusion:**

The median survival time to recovery in this study was found to be optimal. Residency, vaccination status of the child, and analgesic administration were the determinant factors. Paying attention to vaccination coverage, fever management, and pain management as part of the protocol helps reduce the length of hospital stay by facilitating recovery rates among severely malnourished children under five in Ethiopia.

## Background

Severe acute malnutrition (SAM) can be indicated by a weight-for-height/length of <70*%*, visible severe wasting, or the presence of nutritional edema. In children aged 6–59 months, a mid-upper arm circumference (MUAC) of less than 115 mm is also indicative of severe acute malnutrition (1, 2).

It refers to a combination of nutritional disorders that include underweight (mixed), wasting (acute), and micronutrient deficiency (1, 3).

Childhood malnutrition is still a major global health problem, contributing to morbidity, mortality, and risk for disability (4). Globally, the current burden of malnutrition is unacceptably high; since 2018, there have been 150.8 million (22.2%) stunted children (aged 0–59 months) accounts 50.5 million (7.5%) wasted children, and 38.6 million (5.6%) children with overnutrition (5). Across the globe, SAM is responsible for the death of 3.6 million children under the age of five; of these, 45–60% of deaths were due to undernutrition, primarily concentrated in low- and middle-income countries (4, 5). Based on the 2019 Ethiopia Demographic and Health Survey (EDHS), 7% of children under five are wasted, with 1% of these being severely wasted (6). However, there is a regional variation in the overall malnutrition status, ranging from 15% in Addis Ababa to 46% in the Amhara region, compared to the national levels of 10 and 24% for wasting and underweight, respectively (7, 8).

The main causes of nutritional problems across all regions include food insecurity, low dietary diversity, lack of awareness on how to use available resources at home, poor maternal and child feeding practices, inadequate sanitation, poor coordination among sectors, cultural practices, lack of resources, natural disasters, and political instability in the country (9).

Achieving food security, improving nutritional awareness, and advancing the agriculture sector in the country are areas of emphasis that have been fostered as part of the Sustainable Development Goals. Conducting extensive studies on the magnitude and management of severe acute malnutrition by the health sector can provide insight into how to achieve this goal in collaboration with other sectors (10).

Severe acute malnutrition is highly correlated with morbidity, as well as poor growth and development. Therefore, mortality rates remain high among children with severe acute malnutrition in developing nations, including Ethiopia. Children often die because of several factors, such as associated childhood co-morbidities, e.g., diarrhea, pneumonia, and shock, and non-adherence to management protocols by healthcare professionals (11). SAM is one of the top three diet-related non-communicable diseases for children under five, accounting for 8.1% of cases, following pneumonia (15.3%) and neonatal sepsis (20%) among admitted childhood cases in Ethiopia. It is responsible for 25–30% of deaths in many low-income countries, including Ethiopia (12).

The problem of SAM is not only a medical disorder, it also includes social issues. Therefore, successful management of severely malnourished patients requires both medical and social efforts (12). Different studies have shown inconsistencies in the rate of recovery from SAM, ranging from 22.1 to 95.36%. The cure rates have been found to be insufficient across various regions in Ethiopia: Gondar Referral Hospital, 69.2% (3); Bahir Dare Referral Hospital, 58.4% (12); North Gondar zone, 65.3% (13); Nekemte Referral Hospital, 66.8% (14); Southwest Ethiopia, 67.7% (15); Yekatit-12 Hospital in Ethiopia. 81.3% (16); and Debre Markos and Finote Selam Hospitals, 77.9% (17). Over the past 15 years in Ethiopia, the trend of malnutrition has shown a reduction in stunting by 31% and underweight by 39% (12).

Similarly, studies conducted in Ethiopia on the time to recovery from SAM have found it to be in the range of 14–28.8 days (18–23). A shorter recovery time indicates an acceptable level of performance in the treatment and care process.

A variety of factors that predict recovery time have been documented. A high proportion of patients with a long recovery time is attributed to sociodemographic factors, concomitant diseases, and personal and other clinical factors, as well as a healthcare system with limited resources, a lack of trained health personnel, and the inability of the patient or family members to use and afford treatment expenditures (1, 24). In addition, it is also affected by residency, medical comorbidities such as anemia, malaria, dehydration, hypoglycemia, HIV, TB, hyperthermia, hypothermia, and the vaccination status of the child. Though these predictors are assumed to delay the time to recovery, some researchers have reported conflicting results regarding the possible causes of prolonged recovery time (3, 12, 19, 21).

Recovery from SAM remains challenging and insufficient (3). Delays in discharge increase the number of children needing treatment, the cost of medicines, the risk of hospital-acquired infections, and the demand for human resources, thereby imposing an economic burden on resource-poor settings (24, 25). Furthermore, if left untreated, it might result in impairments in physical, intellectual, social, and adaptive behaviors (25). Although Ethiopia has adopted the WHO SAM guidelines and has been working toward the Sustainable Development Goals 2030 under its nutritional strategy to end hunger, achieve food security, improve nutrition, and eliminate deaths due to malnutrition, only limited information exists regarding the outcome of SAM treatment at therapeutic feeding centers. Moreover, SAM remains one of the most common causes of childhood admissions in hospitals, and little is known about recovery time and its predictors in the eastern part of Ethiopia, particularly at Hiwot Fana Comprehensive Specialized Hospital (HFCSH). Therefore, the main objective of this study was to determine the time to recovery and its predictors among 6–59 months old children with SAM treated at the therapeutic feeding center (TFC) of Hiwot Fana Comprehensive Specialized Hospital (HFCSH), who were admitted between 1st September 2019 and 1st March 2024.

## Materials and methods

### Study area and period

The study was conducted in Harar city, located 526 Km far from Addis Ababa, the capital city of the country, in the Harari National Regional State, eastern Ethiopia. According to the current regional health bureau profile, the Harari region has a total of seven hospitals, including four governmental hospitals, two private hospitals, one non-government (Fistula) hospital, eight health centers, 29 private clinics, 26 clinics headed by nurses, and one regional laboratory serving the peoples of the state. This study was conducted at Hiwot Fana Comprehensive Specialized Referral Hospital (HFCSH). Hiwot Fana Hospital is a comprehensive specialized university hospital located in Harar town, and it currently provides various services for more than 5.8 million people in the catchment area.

The study period was from 1st September 2019 to 1st March 2024. We conducted this study during this period of interval to align with the Ethiopian Ministry of Health (EMOH) guidelines for SAM, which have been revised since 2019. The EMOH and stockholders’ SAM report since 2019 identified gaps in diagnosis, management approach, and data management related to pediatric SAM, along with possible recommendations to address these challenges (1).

### Study design

An institution-based retrospective follow-up study was conducted.

### Source population

The source population included all children under five with SAM who were admitted to the therapeutic feeding center at HFCSH.

### Study population

The study population included all children under five with SAM who were admitted to the therapeutic feeding center at HFCSH during the study period.

#### Inclusion criteria

Children under the age of five with a diagnosis of SAM who were admitted for inpatient management according to the national admission criteria for SAM at the HFCSH therapeutic feeding center from 1st September 2019 to 1st March 2024 were included.

#### Exclusion criteria

Children’s medical records or charts with incomplete information (such as unclear diagnosis, anthropometric measurements, and other relevant predictors, including age, date of diagnosis, treatment modality, and the child’s last health condition) and cases transferred in with an unclear date of diagnosis from other institutions were excluded from the study.

### Sample size determination

The sample size was determined using Stata software for the power analysis of the Cox proportional hazards model by considering the following assumptions: a probability of type I error (*α*) of 0.05, a power of 80%, variability of covariates of interest at 0.5, an adjusted hazard ratio (AHR) of 0.49 from a previous study (26), a probability of event of 0.1965, and a proportion of withdrawals of 0.1. Finally, the required sample size was 349. The study participants who met the inclusion criteria were selected using simple random sampling with computer-generated random numbers from the sampling frame.

### Sampling technique and procedure

The study participants were selected from the registration book. The medical records of children who received treatment for SAM from 1st September 2019 to 1st March 2024 were selected. A total of 4,188 children were recorded in the registration book of this referral hospital, of which 349 charts were sampled using a simple random sampling technique with a computer-generated method. Finally, the charts that met the inclusion criteria were reviewed.

#### Dependent variables

The dependent variable was the time to recovery.

#### Independent variables

The independent variables included the following: age of the child, sex of the child, place of residence, educational status of the child, religious status of the family, educational status of the caregiver, marital status of the parents, occupational status of the family, anthropometric measurements (height/recumbent length, weight, and MUAC), comorbidities (TB, HIV, pneumonia, UTI anemia, malaria, diarrhea, vomiting, hypoglycemia, dehydration, hypothermia, septic shock), vaccination status, dermatitis/skin lesions, edema with its degree, routine medications (folic acid, vitamin A supplements, amoxicillin, antipyretics, analgesics, IV antibiotics, and deworming medication), and treatment outcomes of the child. The age of the child in months and weight/body mass index were categorized into groups to align with the existing literature (16).

### Operational definitions

Recovered: the patients were considered recovered when the weight-for-height/length was greater than or equal to 85% of the median WHO growth chart reference, there was an absence of bilateral pitting edema, and there were no medical complications (1, 27). Recovery was documented by a physician in the patient’s medical chart under the discharge summary section.

Time to recovery: the time to recovery was calculated by determining the difference (in days) from the start of the treatment until the child recovered.

Censored: those who were referred/transferred out, were non-responders, defaulted, or died.

Defaulters: those who left the treatment before the child was cured or were lost to follow-up with an unknown status, specifically when the patient discontinued the treatment before recovery.

Event (recovered/cured/censored): being recovered or censored during the study period (1, 3).

Time to event: the time to event was the time interval from the initiation of the treatment until the patient either recovered or was censored during the study period.

Survival time: the duration from the date of diagnosis and the initiation of the treatment to recovery was calculated for each participant.

Time to event: the duration from the initiation of the treatment until the child recovered, measured in days.

Children under five: in this research, children under five included children aged 6–59 months who were admitted to the therapeutic feeding center of HFCSH due to severe acute malnutrition.

### Data collection procedure and tool

The data were collected from the patient charts at HFCSH using a structured data extraction form developed by the investigators after reviewing the literature, including a further inspection of the national SAM management protocol and treatment multichart. The data that were relevant to measure the association between the times to recovery among children under five with SAM were carefully retrieved by two BSc nurses, who were supervised by one senior nurse with a second degree in public health nutrition. The patient records were retrieved using their medical registration numbers (MRNs) identified in the total SAM caseload recorded in the logbook of the registration follow-up form. Then, the medical registration numbers (MRNs) of all patients with SAM were sorted. A computer-generated simple random sampling technique was then applied, ensuring that each of the patients had an equal chance of being selected to be a part of the study. A structured data extraction tool was adapted by considering the study variables, such as socio-demographic factors and personal and clinical predictors from the patients’ charts.

### Data quality control

Training was provided to the data collectors and supervisors regarding the objective and process of data collection by the principal investigator. A pretest was conducted on 5% of the sample size. Then, the pretested data abstraction tool/checklist, which comprised questions designed to measure the relevant variables, was used by the trained data collectors to collect the necessary data from the patients’ medical charts. Data quality was also ensured by designing proper data abstraction tools and through continuous supervision. All collected data were checked for completeness and clarity.

### Data processing and statistical analysis

The collected data were coded, entered, cleaned, and stored in Epi-data version 4.6 and exported to STATA 17.0 statistical software for analysis. Descriptive statistics were presented using frequency tables, Kaplan–Meier (KM) plots, and median survival times. Days were used as a time scale to calculate the time to recovery. The outcome of each participant was dichotomized into censoring or event (recovery). The Kaplan–Meier technique was used to measure the survival experience of different patient groups through survival curves. The log-rank test was conducted to assess significant differences among the survival distributions of the groups for equality. After performing the Cox proportional hazards regression, the model goodness of fit was assessed using Cox–Snell residuals. Assumptions were evaluated with Schoenfeld residuals and graphically using log-minus-log function survival curves. Bivariable analysis was performed to calculate the crude hazard ratio (CHR) and to identify potentially significant independent variables at a *p*-value <0.25 level of significance. The associations between the significant independent variables and the time to recovery were assessed using the multivariable Cox proportional hazards (PH) model. The adjusted hazard ratio (AHR) and 95% confidence interval (CI) for HR were used to test the significance and interpretation of the results. The variables with a *p*-value <0.05 were considered statistically associated with the time to recovery in days.

## Results

### Socio-demographic characteristics *with censoring and event status*

A total of 349 medical records were reviewed; of these, 31 (7.5%) cases were excluded from the study due to pertinent data being missing or replaced with other medical charts in accordance with the predetermined inclusion criteria. As a result, 349 patient medical records were included in the study, resulting in a 100% response rate. The mean age of the study participants was 19.3 months, with a slandered deviation of 14.6.

More than half of the patients were male (56%), and the proportion of recovery among the male patients was 46.4%, which was slightly greater than that among the female participants (37%). The majority of the patients (65%) were from rural areas. Moreover, the proportion of the patients who recovered in rural areas was 52.4%, which was higher than that of the urban area residents (30.9%). The majority of the study participants had uneducated parents as their caregivers (29.5%) (Table 1).

**Table 1:** Sociodemographic-related variables with censoring and event status among the children under five with SAM at the TFC of HFCSH, eastern Ethiopia, 2024 (*n* = 349).

Variable	Category	Event and censored status		Total
		No. of events	No. of censored cases	
Age group in months	6–11	71(20.3%)	16(4.6%)	87(24.9%)
	12–23	82(23.5%)	6(1.7%)	88(25.2%)
	24–35	52(14.9%)	15(4.3%)	67(19.2%)
	36–47	49(14.0%)	14(4.0%)	63(18.1%)
	48–59	37(10.6%)	7(2.0%)	44(12.6%)
Sex	Male	162(46.4%)	33(9.5%)	195(55.9%)
	Female	129(37.0%)	25(7.2%)	154(44.1%)
Resident	Urban	108(30.9%)	14(4.0%)	122(35%)
	Rural	183(52.4%)	44(12.6%)	227(65.0%)
Educational status of the caregiver	Elementary school	71(20.3%)	13(3.7%)	84(24.1%)
	High school	54(15.5%)	7(2.0%)	61(17.5%)
	College graduate	63(18.1%)	16(4.6%)	79(22.6%)
	Uneducated	103(29.5%)	22(6.3%)	125(35.8%)
Marital status of the caregiver	Single	25(7.2%)	4(1.1%)	29(8.3%)
	Married	237(67.9%)	44(12.6%)	281(80.5%)
	Divorced	18(5.2%)	9(2.6%)	27(7.7%)
	Widowed	11(3.2%)	1(0.3%)	12(3.4%)
Religion	Orthodox	50(14.3%)	3(0.9%)	53(15.2%)
	Muslim	214(61.3%)	51(14.6%)	265(75.9%)
	Protestant	25(7.2%)	6(1.7%)	31(8.9%)
Family occupation	Farmer	130(37.2%)	33(9.5%)	163(46.7%)
	Merchant	87(24.9%)	19(5.4%)	106(30.4%)
	Employed	54(15.5%)	6(1.7%)	60(17.2%)
	Unemployed	19(5.4%)	1(0.3%)	20(5.7%)
Child education	KG	10(2.9%)	3(0.9%)	13(3.7%)
	Not started	281(80.5%)	55(15.8%)	336(96.3%)

### Incidence rate of recovery from SAM

Of the 349 study participants, 290 (83.1%) of the patients recovered, with a median time of 17 days, whereas 59 (16.9%) were censored, with a median time of 13 days (95%CI, 9–14). The shortest and longest lengths of follow-up were 6 and 43 days, respectively, and the overall follow-up time in person-days was 2,922.

The overall incidence density recovery rate was 5.7 (95%CI: 4.9–6.6) per 100 person-days of observation. However, the overall censored rate in this study was found to be 7.5 (95%CI: 5.5–10.3) per 100 person-days of observation. The recovery rate among the male and female children with SAM was 5.7 (95%CI, 4.7–7.0) and 5.6 (95%CI, 4.4–7.1) per 100 person-days of observation, respectively, which was nearly comparable for both sexes.

### Treatment protocol-related variables *with censoring and event status*

The treatment protocol for the admitted cases of severe acute malnutrition in this therapeutic feeding center/unit was carried out in accordance with the national guidelines. According to the findings of this study, 335 (96.0%) patients received IV medication during the inpatient treatment. The most frequently administered IV medications were ampicillin and gentamicin; for the patients with a previous history of penicillin administration, ceftriaxone was used.

Of the 349 admitted children with SAM, 336 (96.3%) received formula F-75, while 331 (94.8%) received F-100 milk during their inpatient waiting time. On the other hand, 83 (23.8%) of the total children received blood transfusions. Similarly, 206 (59.0%) children were resuscitated with IV fluids (0.9%sodium chloride or Ringer’s lactate solution with 5% dextrose, with half of each used for 1 h, which was the most commonly used type of fluids). Many of them, 243 (69.6%), had a nasogastric tube (NGT) during the treatment, while only 87 (24.9%) children received deworming medication (Table 2).

**Table 2:** Treatment-related variables with censoring and event status among the children under five with SAM at the TFC of HFCSH, eastern Ethiopia, 2024 (*n* = 349).

Variable	Category	Event and censored status		Total
		No. of events	No. of censored cases	
Intake of F-75	NO	9(2.6%)	4(1.1%)	13(3.7%)
	Yes	281(80.5%)	55(15.8%)	336(96.3%)
Intake of F-100	NO	53(15.2%)	23(6.6%)	76(21.8%)
	Yes	296(84.8%)	35(10.0%)	331(94.8%)
RUTF	NO	119(34.1%)	31(8.9%)	150(43%)
	Yes	171(49.0%)	28(8.0%)	199(57.0%)
Vitamin A	NO	158(45.3%)	28(8.0%)	186(53.3%)
	Yes	132(37.8%)	31(8.9%)	163(46.7%)
Folic acid	NO	232(66.5%)	48(13.8%)	280(80.2%)
	Yes	58(16.6%)	11(3.2%)	69(19.8%)
Deworming medication	NO	217(62.2%)	45(12.9%)	262(75.1%)
	Yes	73(20.9%)	14(4.0%)	87(24.9%)
Oral antibiotics	NO	257(73.6%)	54(15.5%)	311(89.1%)
	Yes	33(9.5%)	5(1.4%)	38(10.9%)
ReSoMal	NO	85(24.4%)	14(4.0%)	99(28.4%)
	Yes	205(58.7%)	45(12.9%)	250(71.6%)
IV fluid	NO	115(33.0%)	28(8.0%)	143(41.0%)
	Yes	175(50.1%)	31(8.9%)	206(59.0%)
IV antibiotics	NO	11(3.2%)	3(0.9%)	14(4.0%)
	Yes	279(78.0%)	56(16.0%)	335(96.0%)
Blood transfusion	NO	226(64.8%)	40(11.5%)	266(76.2%)
	Yes	64(18.3%)	19(5.4%)	83(23.8%)
Analgesics^*^	NO	169(48.4%)	35(10.0%)	332(95.1%)
	Yes	121(34.7%)	24(6.9%)	145(41.5%)

*The majority of the patients used ibuprofen as an analgesic. However, in a few cases, requests for paracetamol were also noted.

### *Comorbidity-*related variables *with censoring and event status*

Regarding comorbidity, all admitted children with SAM had a history of comorbid illness, and the most common comorbidity was pneumonia 264 (75.6%), followed by fever/hyperthermia 251 (71.9%), diarrhea/AGE 246 (70.5%), vomiting 222 (63.6%), dehydration 183 (52.4%), and anemia 137 (39.3%). The proportion of the patients who recovered was higher among those with no history of hypothermia (63.3%) compared to those with such a history (19.8%) (Table 3).

**Table 3:** Comorbid illness/medical complication-related variables with censoring and event status among the children under five with SAM at the TFC of HFCSH, eastern Ethiopia, 2024 (*n* = 349).

Variables	Category	Event and censored status		Total
		No. of event	No. of censored cases	
Fever	NO	83(23.8%)	15(4.3%)	98(28.1%)
	Yes	207(59.3%)	44(12.6%)	251(71.9%)
Hypothermia	NO	221(63.3%)	50(14.3%)	271(77.7%)
	Yes	69(19.8%)	9(2.6%)	78(22.3%)
Pneumonia	NO	77(22.1%)	8(2.3%)	85(24.4%)
	Yes	213(61.0%)	51(14.6%)	264(75.6%)
Urinary tract infection (UTI)	NO	258(73.9%)	38(10.9%)	296(84.8%)
	Yes	32(9.2%)	21(6.1%)	53(15.2%)
Vomiting	NO	108(30.9%)	19(5.4%)	127(36.4%)
	Yes	182(52.1%)	40(11.5%)	222(63.6%)
Upper respiratory tract infection (URTI)	NO	294(84.2%)	30(8.6%)	324(92.8%)
	Yes	15(4.3%)	10(2.9%)	25(7.2%)
Acute gastroenteritis (AGE)	NO	84(24.1%)	19(5.4%)	103(29.5%)
	Yes	206(59.0%)	40(11.5%)	246(70.5%)
Dehydration	NO	136(39.0%)	30(8.6%)	166(47.6%)
	Yes	154(44.1%)	29(8.3%)	183(52.4%)
Shock	NO	250(71.6%)	45(12.9%)	295(84.5%)
	Yes	40(11.5%)	14(4.0%)	54(15.5%)
Tuberculosis (TB)	NO	288(82.5%)	55(15.8%)	343(98.3%)
	Yes	2(0.6%)	4(1.1%)	6(1.7%)
HIV	NO	287(82.2%)	57(16.3%)	344(98.6%)
	Yes	2(0.6%)	3(0.9%)	5(1.4%)
Malaria	NO	282(80.8%)	55(15.8%)	337(96.6%)
	Yes	8(2.3%)	4(1.1%)	12(3.4%)
Fungal infection	NO	278(79.7%)	44(12.6%)	322(92.3%)
	Yes	14(4.0%)	13(3.7%)	27(7.7%)
Measles	NO	282(80.8%)	57(16.3%)	339(97.1%)
	Yes	8(2.3%)	2(0.6%)	10(2.9%)
Anemia	NO	177(50.7%)	35(10.0%)	212(60.7%)
	Yes	113(32.4%)	24(6.9%)	137(39.3%)
Dermatosis	NO	231(66.2%)	48(13.8%)	279(79.9%)
	Yes	59(16.9%)	11(3.2%)	70(20.1%)

### Median survival time to recovery

The estimated median survival time to recovery was 17 days, with an interquartile range of 16–18 days. Meanwhile, the median time for the censored cases was found to be 13 days (95%CI: 9–14), which significantly suggested that almost all censored cases, such as defaulters and deaths, occurred within 2 weeks of admission. However, the median survival time to recovery among the children with SAM varied across different categories of predictors. For example, the median survival time for the children aged 6–11 months was 16 days, while for those aged 12–23, 24–35, 36–47, and 48–59 months, it was 17, 16, 17, and 19 days, respectively (Table 4).

**Table 4:** Comparisons of recovery among the children under five with SAM at the TFC of HFCSH, eastern Ethiopia, 2024 (*n* = 349).

Variable	Category	Test of equality over groups		Log-rank		
		Median survival time (days)	Mean survival time (days)			
				*X* ^2^	DF	*P*-value
Age group in months	6–11	16	16.4	5.12	4	0.2756
	12–23	17	17.1			
	24–35	16	16.9			
	36–47	17	19.5			
	48–59	19	18.5			
Sex	Male	17	17.4	0.19	1	0.6609
	Female	17	17.8			
Resident	Urban	18	18.4	1.38	1	0.2403
	Rural	17	17.2			
Education status of children	KG	21	22.5	3.36	2	0.0668
	Not started	17	17.4			
Type of admission	New	17	17.6	<0.001	1	0.9502
	Readmission	15	17.4			
Edema	NO	17	16.8	3.29	1	0.0699
	Yes	17	18.5			
EBF	NO	17	18.9	0.02	1	0.8889
	Yes	17	17.9			
Vaccination status	Fully vaccinated	15	15.9	6.77	2	0.0338
	Partially vaccinated	18	18.9			
	Not at all	17	18.4			
F-75	NO	12	13.3	2.46	2	0.1170
	Yes	17	17.7			
F-100	NO	16	16.5	3.46	1	0.0627
	Yes	17	18.4			
RUTF	NO	16	16.0	7.96	1	0.0048
	Yes	17	18.6			
Oral antibiotic	NO	17	17.5	0.85	1	0.3569
	Yes	17	19.0			
IV antibiotics	NO	17	23.3	2.52	1	0.1126
	Yes	17	17.5			
Folic acid	NO	16	17	6.54	1	0.0105
	Yes	19	20.4			
Vitamin A	NO	17	17.1	1.07	1	0.3010
	Yes	17	18.2			
Analgesics	NO	17	18.6	3.82	1	00.0507
	Yes	16	16.6			
ReSoMal	NO	17	18.0	0.62	1	0.4293
	Yes	16	17.4			
Fever	NO	17	17.3	1.68	1	0.1944
	Yes	18	18.6			
Dehydration	NO	16	16.9	0.47	1	0.4950
	Yes	17	18.1			
Shock	NO	16	16.2	1.06	1	0.3024
	Yes	17	17.8			
Pneumonia	NO	16	17.5	0.00		0.9754
	Yes	17	17.7			
Acute gastro enteritis	NO	17	17.4	0.01	1	0.9248
	Yes	17	17.6			
Dermatosis	NO	17	17.1	5.71	1	0.0169
	Yes	19	19.7			
Anemia	NO	17	17.4	0.07	1	0.7963
	Yes	17	17.7			

*X2, chi-square; DF, degree of freedom; KG, kindergarten.*

### Survival estimates for the time to recovery

**Figure 1:**
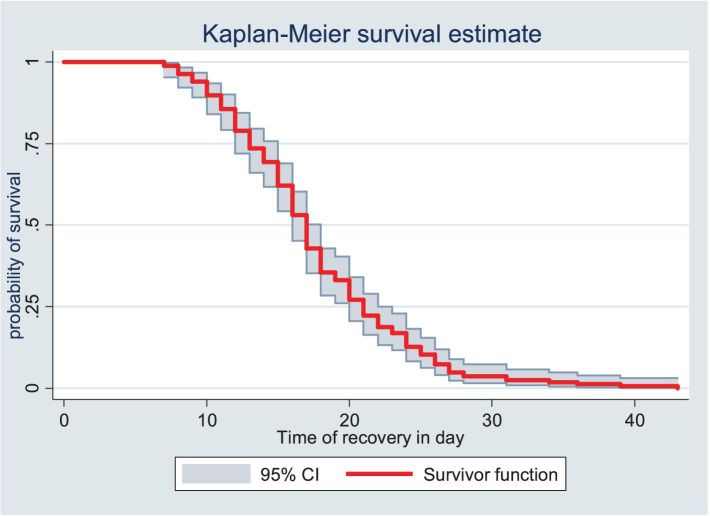
Kaplan–Meier survival estimate of the time to recovery among the children under five with SAM at the TFC of HFCSH, eastern Ethiopia, 2024 (*n* = 349).

**Figure 2:**
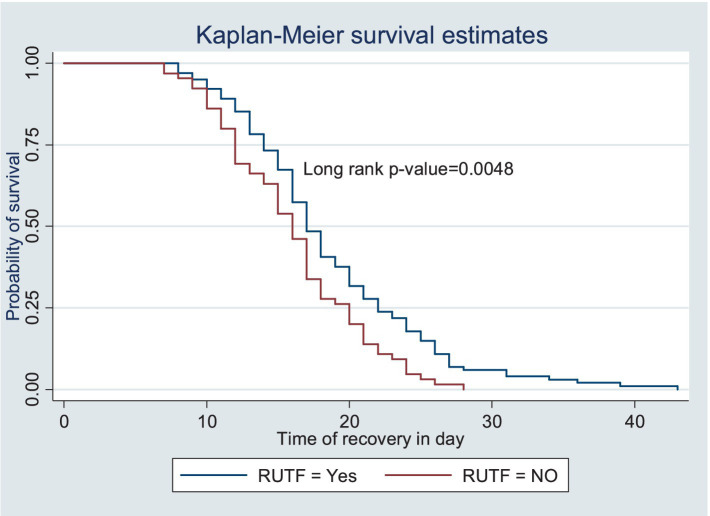
Kaplan–Meier survival estimate of the time to recovery among the children under five with SAM receiving RUTF at the TFC of HFCSH, eastern Ethiopia, 2024 (*n* = 349).

**Figure 3:**
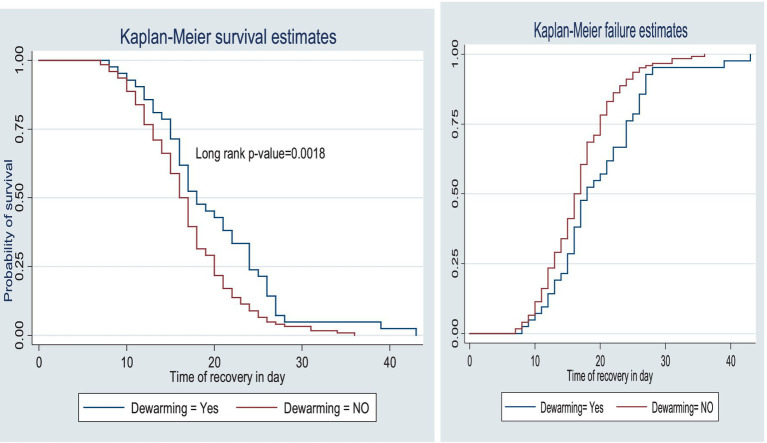
Survival and hazard functions of deworming medication over time (in days) at the TFC of HFCSH, eastern Ethiopia, 2024 (*n* = 349).

**Figure 4:**
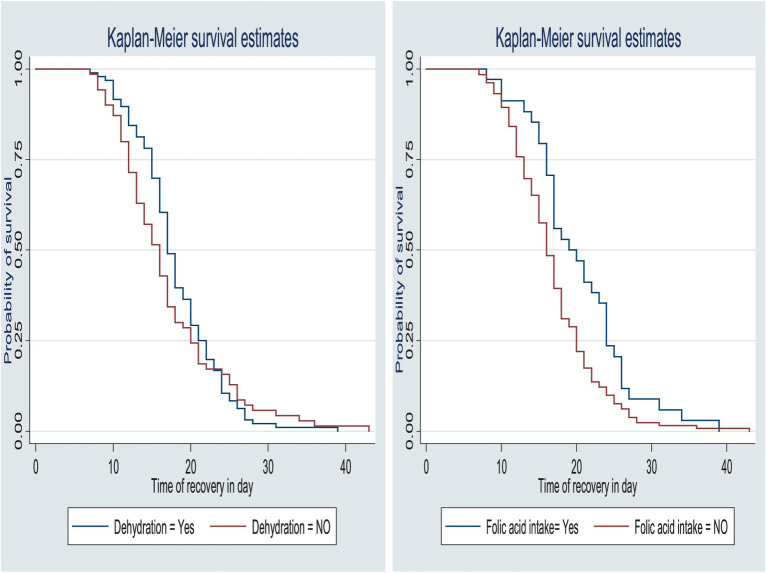
Survival function by dehydration status and folic acid intake for the time to recovery among the children under five with SAM at the TFC of HFCSH, eastern Ethiopia, 2024 (*n* = 349).

The survival status of the children with SAM was estimated using the Kaplan–Meier survival curve. The curve tended to decrease rapidly within the first 2 weeks, indicating that the majority of the children achieved recovery during this time (Figure 1). The survival estimates of the patients varied in relation to different predictors (Figures 2–4). The Kaplan–Meier survival curve showed that the patients who received RUTF supplementation, deworming medication, and folic acid intake had a satisfactory survival experience by achieving recovery early. The figure also showed that the direct chance of being cured/recovered increased for both groups as the duration of the treatment increased.

**Figure 5:**
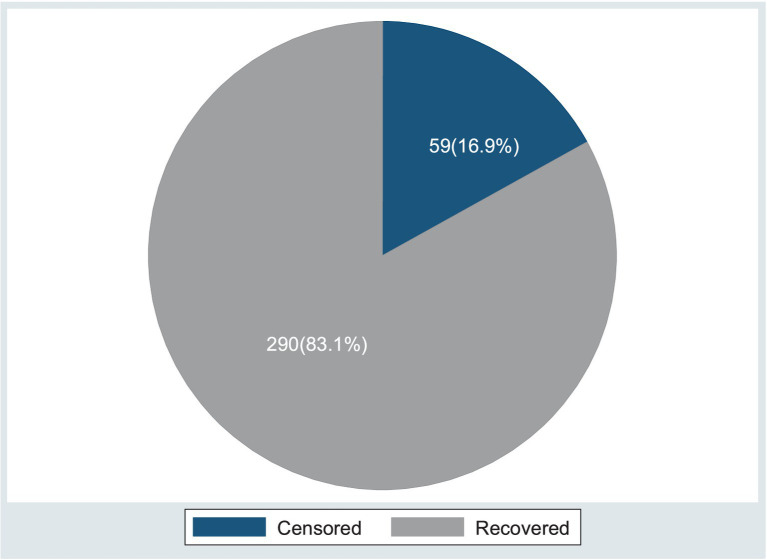
Present status of the event (recovery and censored) among the children under five with SAM at the TFC of HFCSH, eastern Ethiopia, 2024 (*n* = 349).

The proportions in the treatment outcomes of the entire cohort were within the acceptable range according to the international reference standards for SAM outcome indicators in children in therapeutic feeding units, which state that there should be > 75% recovery, < 10% death, and <15% defaulters, with a mean length of stay of 28 days (Table 5 and Figure 5).

**Table 5:** Performance indicators for the children under five with SAM at the HFCSH TFU compared with the sphere project based on 2016 standards (*N* = 349).

Performance indicators	HFCSH		Sphere project reference value	
	Frequency	Percentage	Acceptable	Alarming
Recovery	290	83.1%	>75%	<50%
Defaulter	32	9.2%	<15%	>25%
Death	17	4.9%	<10%	>15%
Non-responder	7	2.0%	–	–
Transferred out	3	0.9%	–	–
Average length of stay	17.05 days		<28 days	>41 days

### Comparison of survival experience

The log-rank test was used to assess the differences in the equality of the survival distribution among the diverse groups. The median survival time for recovery among the urban resident participants was relatively longer (18 days) compared to the rural residents (17 days), and the survival time was significantly different among the residents [*X*^2^(2) = 1.38; *p* = 0.2403]. Regarding vaccination status, the children who were fully vaccinated had a shorter recovery time (15 days) compared to those who were not vaccinated according to their age (18 days). The long rank test was statistically significant [*X*^2^(1) = 6.77, *p* = 0.0338]. In contrast, the median survival time to recovery among the patients in the age group of 6–11 months was shorter (16 days) compared to those in the age groups of 12–23 months (17 days), 36–47 months (17), 48–59 months (19 days). However, the long rank test was not statistically significant [*X*^2^(1) = 5.12, *p* = 0.2756] (Table 4).

The median survival time to recovery from SAM was shorter among the patients with no history of dermatosis as a comorbid illness (17 days) compared to those who had it (19 days), with statistically significant differences among the groups [*X*^2^(1) = 5.71, *p* = 0.0169]. However, no statistically significant differences were observed for sex, type of admission, EBF, Vitamin A, ReSoMal, and a history of anemia in determining the time to recovery (Table 4).

### Results of the multivariable cox proportional hazards model

**Figure 6:**
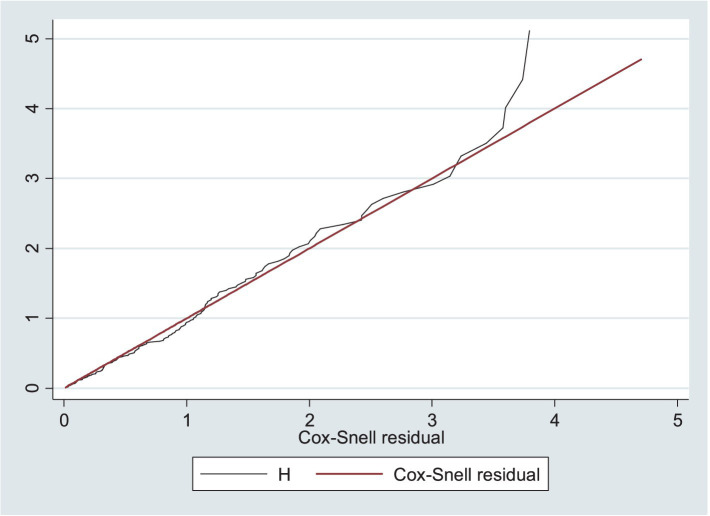
Model goodness of fit assessed using Cox–Snell residuals among the children under five with SAM at the TFC of HFCSH, eastern Ethiopia, 2024 (*n* = 349).

The goodness of fit was assessed using Cox–Snell residuals by plotting them against the cumulative hazard function. As the residuals follow a unit exponential distribution or a linear line through the origin with a unit gradient, this indicates a well-fitted model to the observed data points and expected values (Figure 6).

**Figure 7:**
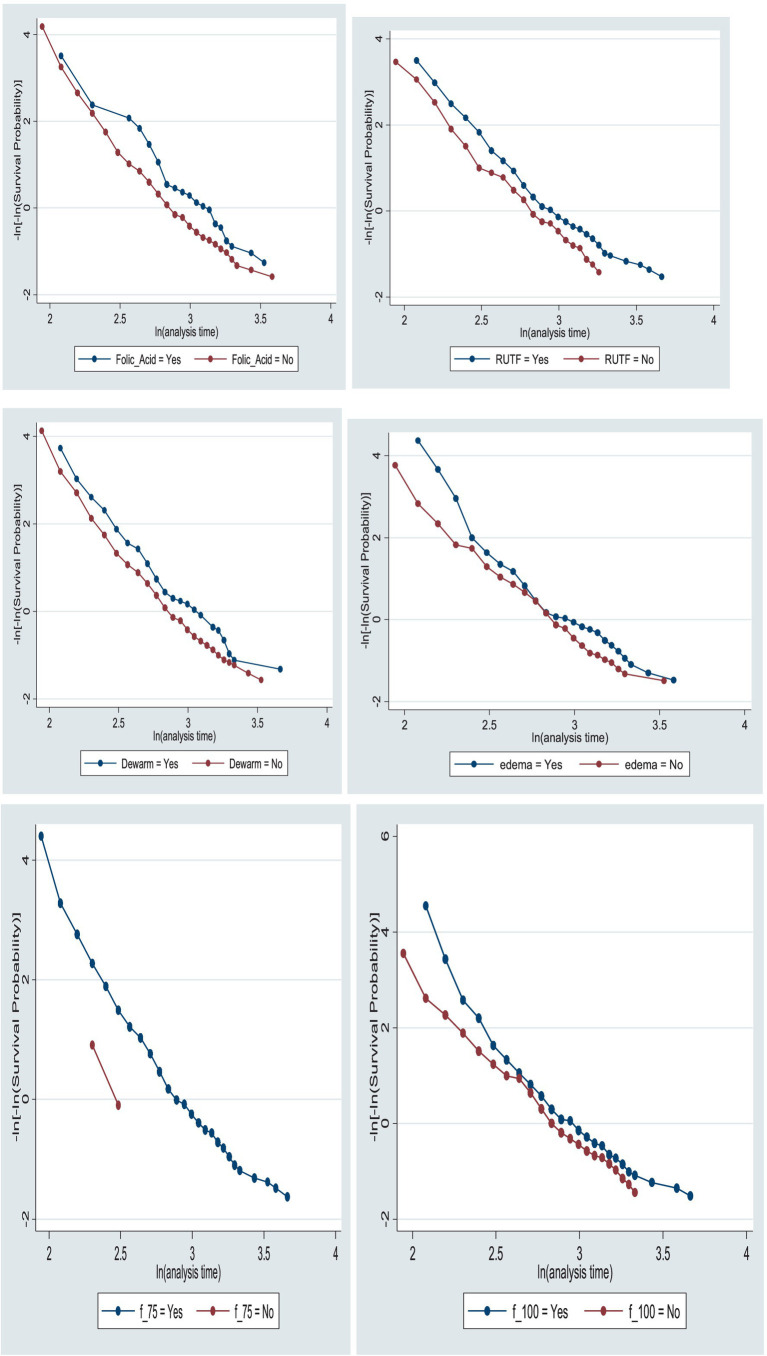
Log-minus-log function survival curves of folic acid intake, RUTF, deworming medication, edema, and F-75 and F-100 intakes for the time to recovery among the children under five with SAM at TFC of HFCSH, eastern Ethiopia, 2024 (*n* = 349).

The proportional assumption of the Cox proportional hazards model was tested using Schoenfeld residuals test/the global test (0.5368) and graphically with the log-minus-log function survival curves in Stata version 17.0. The survival curve appeared parallel throughout the study time, indicating equitable fitting to the proportional hazard assumption (Figure 7).

The presence of interaction among the independent variables was assessed using the multicollinearity test, which showed no significant interaction. This was confirmed by the value of each variable’s variance inflation factor (VIF), which was less than seven (mean value of VIF = 2.62).

The independent variables, such as age, weight, and height/length of the child, residence, client educational status, vaccination status, edema, appetite test, fever, dermatosis, F-75, F-100, RUTF, folic acid intake, IV antibiotics, deworming medication, and analgesics, were significantly associated with the time to recovery at a significance level of less than 0.25, as determined in the bivariable analysis. However, only residence, vaccination status, and analgesic administration were found to be significantly associated with the time to recovery in the multivariable Cox proportional hazards model, at a significance level of less than 5%.

Consequently, after adjusting for other predictors, the hazard of recovery among the rural resident children increased by 2.072 compared to the urban resident children (AHR = 2.072; 95%CI = 1.336–3.215). The rate of recovery among the patients who were fully vaccinated was 1.848 times greater than that of the patients who were not vaccinated at all according to their age (AHR = 1.848; 95%CI = 1.162–2.939).

However, the hazard of recovery among the patients with no analgesic administration was 31.5% lower compared to the patients who received analgesics during their admission (AHR = 0.685; 95%CI = 0.472–0.995). This means that the time needed to recover among the patients with no documented evidence of analgesic administration for the management of fever and pain in the child was significantly longer compared to the patients who received analgesics (Table 6).

**Table 6:** The results of the bivariable and multivariable Cox proportional hazards models among the children under five with SAM at the TFC of HFCSH, eastern Ethiopia, 2024 (*n* = 349).

Variable	CHR(95%CI)	AHR(95% CI)	*P*-value
Age of the child	0.985(0.975–0.995)*	1.003(0.981–1.025)	0.786
Weight of the child	0.916(0.867–0.968)*	0.952(0.839–1.079)	0.445
Height/length of the child	0.972(0.957–0.986)*	0.996(0.962–1.031)	0.804
Residence			
Urban^®^			
Rural	1.146(1.129–1.645)*	2.072 (1.336–3.215)	0.001**
Educational status			
KG^®^			
Not started	1.843(1.701–3.7703)*	1.413(0.569–3.504)	0.456
Vaccination status			
Not vaccinated®			
Partially vaccinated	0.904(0.608–1.344)	1.164(0.721–1.877)	0.534
Fully vaccinated	1.406 (1.084–2.009)*	1.848 (1.162–2.939)	0.009**
Presence of edema			
Yes^®^			
NO	1.309(1.0592–1.785)*	1.187(0.751–1.876)	0.462
Appetite test			
Pass^®^			
Failed	1.3322 (1.013–2.182)*	1.039(0.594–1.819)	0.893
Fever			
Yes^®^			
NO	0.804(0.566–0.944)*	0.820 (0.536–1.255)	0.361
Dermatosis			
Yes^®^			
NO	1.571(1.053–2.344)*	1.567(0.944–2.602)	0.082
F-75			
Yes^®^			
NO	2.338(1.741–7.37667)*	1.323(0.357–4.904)	0.676
RUTF			
Yes^®^			
NO	1.540(1.117–2.123)*	1.243(0.826–1.869)	0.297
Folic acid intake			
Yes^®^			
NO	1.586(1.083–2.322)*	1.235(0.740–2.061)	0.419
IV antibiotics			
Yes^®^			
NO	0.422(0.131–0.858)*	0.568(0.145–2.220)	0.416
Deworming medication			
Yes^®^			
NO	1.573(1.096–2.259)*	0.978(0.623–1.537)	0.925
Analgesics			
Yes^®^			
NO	0.751(0.553–1.021)*	0.685(0.472–0.995)	0.047**

CHR, crude hazard ratio; AHR, adjusted hazard ratio; Rx, treatment, ®, reference group, and * and ** indicate statistically significant variables within the bivariable and multivariable Cox proportional hazards model, respectively.

## Discussion

The study aimed to estimate the time to recovery and identify its predicting factors among children under 5 years of age with SAM at the therapeutic feeding center of HFCSH. Among the 349 participants included in this study, 290 (83.1%) recovered, 32 (9.2%) defaulted, 17 (4.9%) died, three (0.9%) transferred out, and seven (2.0%) were recorded as non-responders during the management process. The minimum and maximum recovery times for the entire cohort were 6 and 43 days, respectively. The proportions of the treatment outcomes of the entire cohort were within the acceptable range according to the international reference standards for SAM outcome indicators in children in therapeutic feeding units, which state there should be > 75% recovery, < 10% death, and <15% defaulters, with a mean length of stay of 28 days. The median survival time to recovery was 17 days, with the overall incidence rate of recovery being 5.7 (95%CI: 4.9–6.6) per 100 person-days of observation.

Although no clear cut-off point has been established about when to consider recovery, most clinical experts expect complete recovery within 4 weeks of admission for first-diagnosed SAM, provided that treatment is initiated simultaneously and carried out according to the nationally recommended SAM treatment guidelines, with strict monitoring and evaluation using a multichart approach as well (28). Based on the follow-up measurement tool/multichart used by the hospital, the findings of this study indicate a suboptimal time to recovery from SAM among children under five.

The median time to recovery in this study was consistent with the findings of studies conducted in Bahir Dar city (16.05 days) (19), Jimma University (17.4 days) (29), Hawassa University (17 days) (20), Yirgalem General Hospital (18 days) (30), and selected government health institutions in the Amhara region (16 days) (22). However, there have been studies conducted in Bahir Dar, Felege Hiwot Referral Hospital (24 days) (18), Southern Ethiopia (29 days) (31), Sekota Hospital (32), Jimma (19 days) (21), Tigray region (49 days) (33), and Dire Dawa (61 days) (34) with longer median times to recovery than the current study. The variation in recovery time among these study settings might be due to differences in the severity of the cases admitted, the quality services delivered, the availability of supplies used for all types of care, the level of the healthcare system, and the timings of the study conducted (35).

The findings of our study showed a longer recovery time than those of studies conducted in Yekatit-12 Hospital (median time,15 days) (16), Pawe General Hospital (14 days) (36), Sekota Hospital (10 days) (32), Woldia (13 days) (37), and eastern Ethiopia (13 days) (38). This finding also indicated a longer median time to recovery compared to a study conducted outside of Ethiopia (6 days) (39). The reasons for such significant inconsistencies could be differences in socioeconomic status, the quality of care provided to children, health-seeking behavior, and the availability of therapeutic foods and medications (40). Another justification for the disparity could be differences in study settings as some studies were conducted in referral and specialized hospitals where children with the most severely deteriorated cases of SAM are referred (38).

Regarding the predictors, the study participants from rural areas were found to be significantly associated the variables that determined the time to recovery. The study showed that the time needed to recover from SAM was shorter among the patients from rural areas compared to the patients from urban areas, indicating that for the children from rural areas, the rate of recovery increased twofold. This might be due to community health workers and nutrition programs being more accessible in rural settings, leading to earlier detection and treatment of malnutrition (1). However, this finding is in contradiction to the findings reported from Shebedino, southern Ethiopia, which stated that walking for more than 1 h to receive treatment is less likely to result in recovery (41). This could be due to differences in study settings, demographic characteristics, clinical presentations, and other circumstances (inpatient vs. outpatient). In this study, the number of the female participants from rural areas was almost twice that of the male participants, which was reported as one of the predictor variables in the study conducted in the Amhara region (22). Moreover, in this study, the number of the non-edematous/marasmus children was greater than that of the edematous children, with the presence of edema being found significant in previously reported studies (23, 37, 42). Another justification may be the environmental triggering effect of stimulation; rural children may be more stimulated by the hospital setting than urban children, as supported by the study conducted in Jimma (21).

Vaccination status of the children was also significantly associated with the time to recovery; specifically, the fully vaccinated children were approximately twice as likely to recover compared to the children who were not vaccinated at all according to their age. This may be due to the fact that the immune systems of unvaccinated children cannot defend against major childhood diseases, such as pneumonia, TB, diarrhea, meningitis, measles, and other vaccine-preventable diseases. A compromised immune system becomes more pronounced among severely malnourished children. As a result, a child experiencing alterations in nutritional status is more likely to have other comorbidities, which, in turn, prolong recovery time. The finding is supported by the findings of the studies conducted in Pawe General Hospital, Northwest Ethiopia (36), Gubalafto Wereda, North Wollo Zone (43, 44), Bahir Dar (18), and Jimma (21). This finding also aligns with results reported from other developing countries (45). Furthermore, the impact of severe acute malnutrition worsens as innate and adaptive immune dysfunction leads to immune dysregulation (45, 46). However, studies reported from North Shoa and Enderta in the Tigray Region did not show any association between vaccination status and recovery time from SAM among children under five (33, 47). The reason for this variation could be differences in the healthcare system and sample size.

The rate of recovery decreased by 31.5% for the children who did not receive analgesics for pain management, meaning that the absence of analgesic administration was 0.685 times less likely to be associated with the recovery rate. This finding aligns with another study reported so far (48). Severe acute malnutrition (SAM) often leads to medical complications due to metabolic disturbances and compromised immunity; these complications can include pain (12). Several authors have attempted to describe the association between nutritional recovery and pain. They found that pain can lead to depression, which often occurs in immunocompromised patients and is seen as one of the most common causes of weight loss and malnutrition (49). Pain also negatively affects the cognitive status of a person by impairing attention, deliberation, and mentation, which can result in lower appetite and further lead to weight alteration (49–51). This implies that pain management in children with severe acute malnutrition (SAM) is crucial for their overall well-being and recovery.

Overall, this study can have positive implications for clinical care, health service management, and research in the areas of nutrition, malnutrition, and metabolism. Clinically, healthcare workers can identify predictors associated with the time to recovery among children with SAM in a clinical setting. Healthcare managers can access current evidence about the time it takes for patients to reach an optimal recovery state and take remedial action to strengthen service delivery by clinicians. Researchers can also be motivated to conduct further studies in this area using more advanced research models with larger sample sizes.

## Conclusion and recommendations

The median survival time to recovery in this study was found to be suboptimal. Residency, vaccination status of the child, and analgesic administration were the predictors. Paying attention to vaccination coverage, fever management, and pain management as part of the protocol helps reduce the length of hospital stay by facilitating recovery rates among severely malnourished children under five in Ethiopia. However, the impact of residing in urban areas on recovery needs further research using different models in the future.

### Strength of the study

One of the strengths of this study is that it provides current evidence on the predictors associated with the time to recovery among children with SAM, which, to the best of my knowledge, has not previously been documented in the study area in recent times.

### Limitation of the study

As the data were collected from medical records, variables such as parental socio-economic factors could not be addressed through chart review, which might have affected the outcome of the study.

## Data Availability

The original contributions presented in the study are included in the article/supplementary material, further inquiries can be directed to the corresponding author.
